# Port site hernia repair using the VersaOne™ Fascial Closure System: a case report

**DOI:** 10.1093/jscr/rjaa552

**Published:** 2020-12-26

**Authors:** Emi Hagui, Masahiro Kimura, Takeyasu Katada, Takaya Nagasaki, Seiichi Nakaya, Yuki Eguchi, Saburo Sugita, Misato Sawai, Yoshiyuki Kuwabara, Akira Mitsui

**Affiliations:** Department of Surgery, Nagoya City East Medical Center, Nagoya, Aichi, Japan; Department of Surgery, Nagoya City East Medical Center, Nagoya, Aichi, Japan; Department of Surgery, Nagoya City East Medical Center, Nagoya, Aichi, Japan; Department of Surgery, Nagoya City East Medical Center, Nagoya, Aichi, Japan; Department of Surgery, Nagoya City East Medical Center, Nagoya, Aichi, Japan; Department of Surgery, Nagoya City East Medical Center, Nagoya, Aichi, Japan; Department of Surgery, Nagoya City East Medical Center, Nagoya, Aichi, Japan; Department of Surgery, Nagoya City East Medical Center, Nagoya, Aichi, Japan; Department of Surgery, Nagoya City West Medical Center, Nagoya, Aichi, Japan; Department of Surgery, Nagoya City West Medical Center, Nagoya, Aichi, Japan

**Keywords:** port site hernia, abdominal incisional hernia, port site closure, laparoscopic herniorrhaphy

## Abstract

The use of laparoscopic surgery has become widespread in recent years. One of its complications is port site hernia (PHS). It can be difficult to close the fascia at the time of laparoscopy, especially in obese patients, and there is a risk of herniation through a fascial defect with incomplete closure. It is important to ascertain closure of the defect when repairing PHS to prevent recurrence. We report a 47-year-old woman who developed a PHS at the superior aspect of the umbilicus. We repaired the defect using the VersaOneTM Fascial Closure System with laparoscopic guidance. This system allows the port site to be reliably closed while observing the suture from the abdominal cavity. The incision is the same size as a port site. If the abdominal wall is thick and the PHS has a diameter of ~10 mm, this method is considered to be indicated, regardless of the site.

## INTRODUCTION

As laparoscopic surgery has become an increasingly common procedure, reports of port site hernias (PHS) have been increasing. Although the laparoscopic approach creates smaller incisions than laparotomy, it is more difficult to close the fascia, especially when the abdominal wall is thick. There is a risk for future herniation through any remaining fascial defect. Repairing PHS requires certainty of complete fascial closure to prevent recurrence. We repair PHS using the VersaOne™ Fascial Closure System with laparoscopic guidance, with good results.

## CASE REPORT

**Figure 1:**
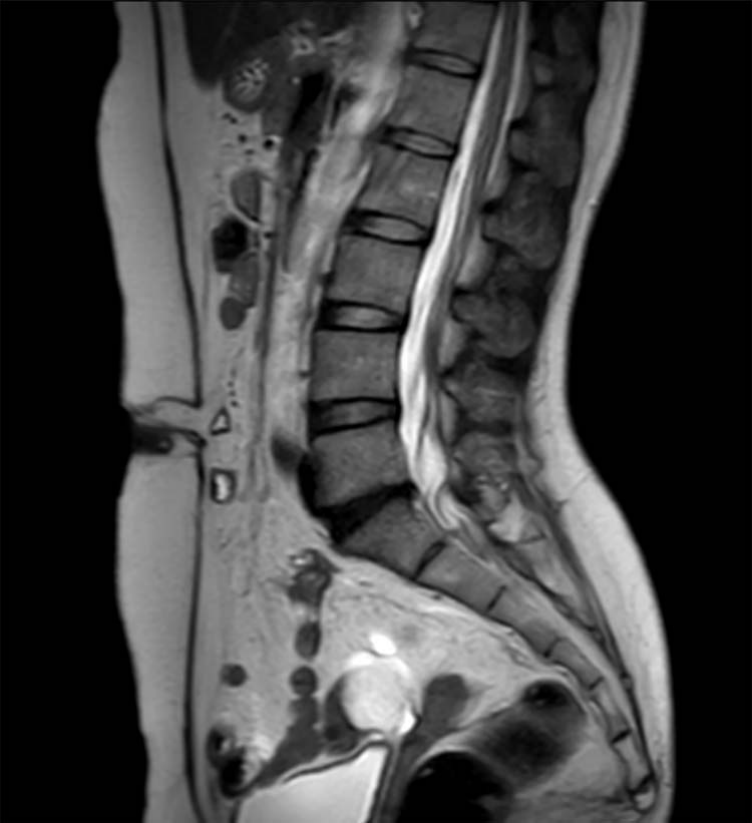
MRI sagittal view of the port site hernia.

**Figure 2:**
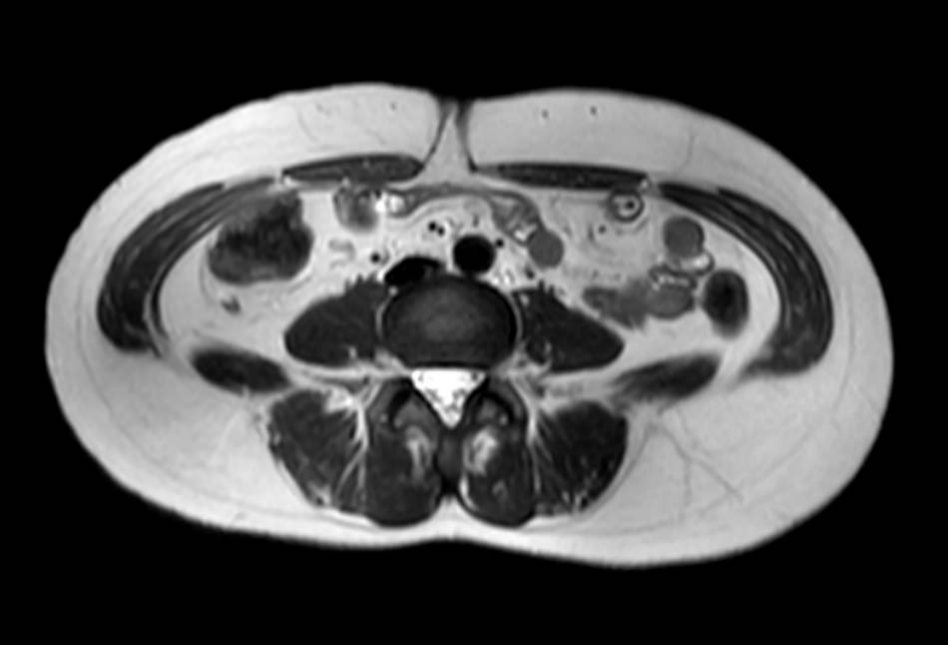
MRI axial view of the port site hernia.

A 47-year-old woman was referred to us by her obstetrician. She developed a PHS after laparoscopic total hysterectomy with right oophorectomy performed 3 years previously. She reported pain with intermittent bulging on the superior aspect of the umbilical region. Clinical examination revealed a single hernia orifice at that location. Her height was 161.1 cm, with a weight of 60.1 kg, yielding a body mass index of 23.16 kg/m^2^. Her medical history included uterine fibroids and ovarian cysts. She had no history of smoking. Magnetic resonance imaging (MRI) revealed protrusion of adipose tissue at the upper side of the umbilical region, through a fascial defect measuring 9 × 13 mm; diastasis recti was also noted (Figs 1 and 2).

**Figure 3:**
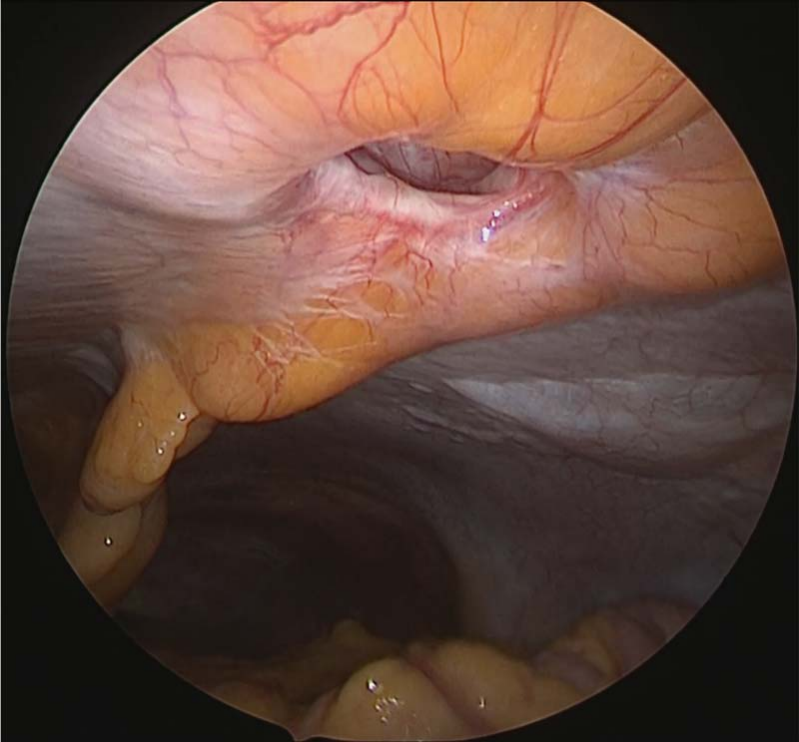
Hernia orifice.

**Figure 4:**
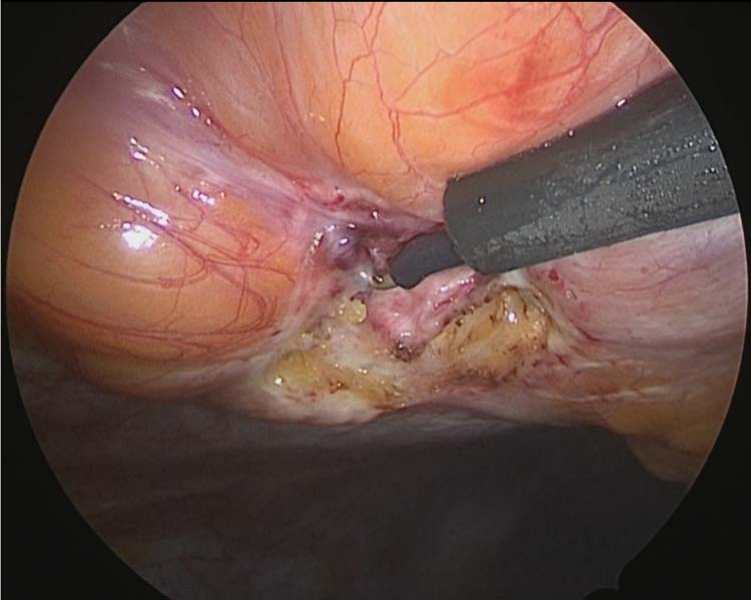
Exposure of the muscle layer around the hernia orifice.

**Figure 5:**
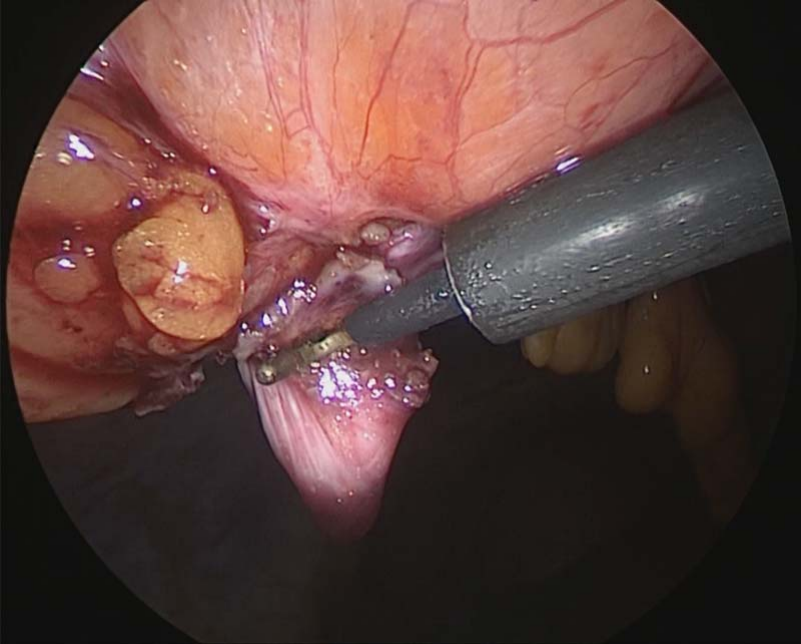
The hernia sac is excised.

**Figure 6:**
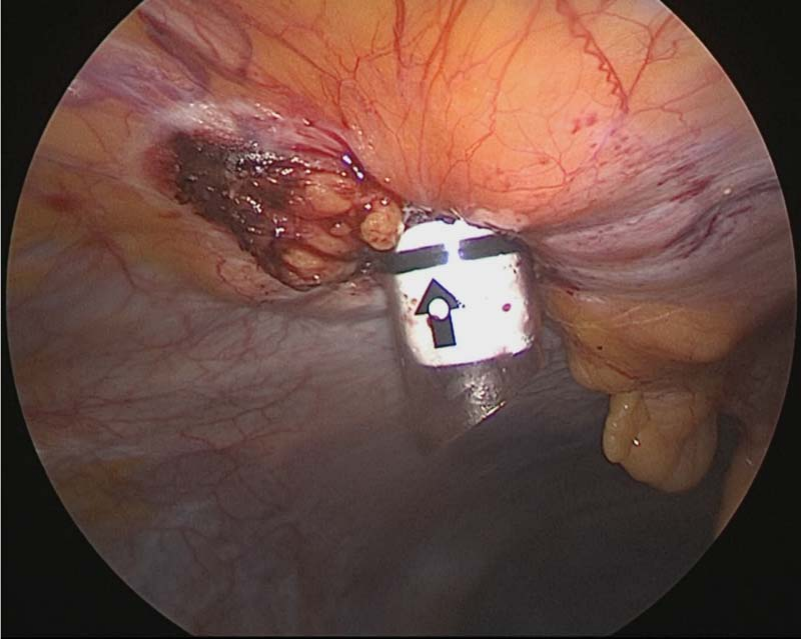
A 12-mm trocar is inserted into the hernia orifice.

**Figure 7:**
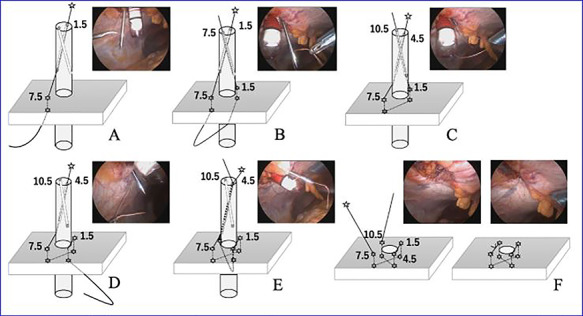
The VersaOneTM Fascial Closure System in use.

**Figure 8:**
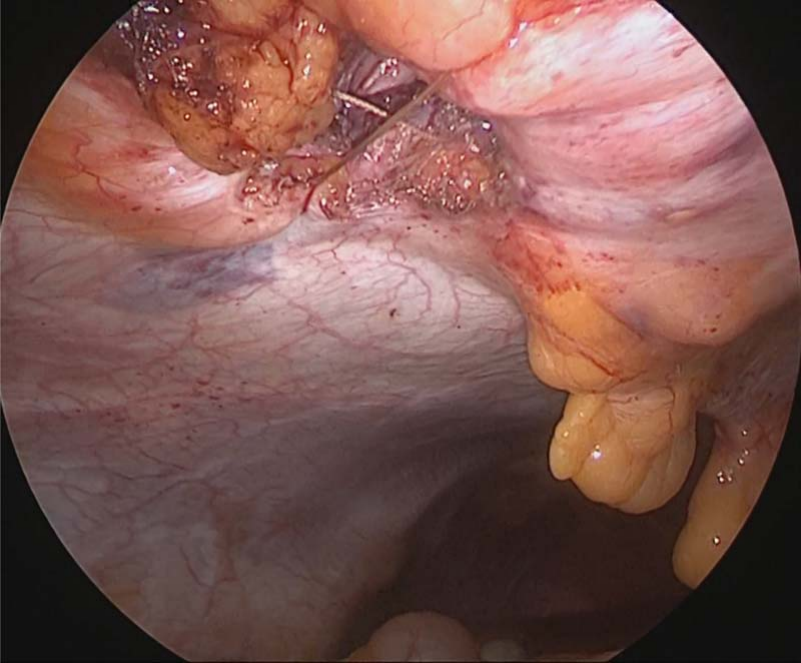
Completed Z suture.

Under general anesthesia, with the patient in the lithotomy position, a 5-mm trocar was inserted into the lower right abdomen using the optical method. Observation of the abdominal cavity revealed a hernia orifice with a diameter of ~1 cm at the upper aspect of the umbilicus (Fig. 3). Two additional 5-mm trocars were inserted into the left side of the abdomen. The hernia sac was significant scarring. We then made an incision around the hernia orifice using a hook-type electrocautery electrode to expose the muscle layer (Fig. 4). The hernia sac was pushed from the surface of the body using a pean, inverted into the abdominal cavity, and excised as much as possible using electrocautery (Fig. 5). At that point, we employed the VersaOne™ Fascial Closure System to close the fascia and peritoneum at the port site.

This system uses a 12-mm trocar, through which an inner cylinder can be inserted to use as a guide for suture closure. The guide contains two diagonal channels. As shown in Fig. 7, the suture is inserted from one side and pulled out from the other side.

We inserted the system’s 12-mm trocar into the hernia orifice (Fig. 6) and inserted the guide through the trocar. A suture was inserted into the abdominal cavity from the 1:30 position using a passer, and the passer was reinserted at the 7:30 position to pull out the suture. The trocar was then rotated 90° counterclockwise. The suture that now emerged from the 10:30 position was grasped with a passer and returned once more to the abdominal cavity, where it was grasped with the passer now inserted from the 4:30 position and pulled back to the outside of the wound (Fig. 7). The trocar was removed, and after confirming that the Z suture was completed, the thread was ligated (Fig. 8). The operative time was 1 h and 20 min, and the amount of bleeding was 5 ml.

**Figure 9:**
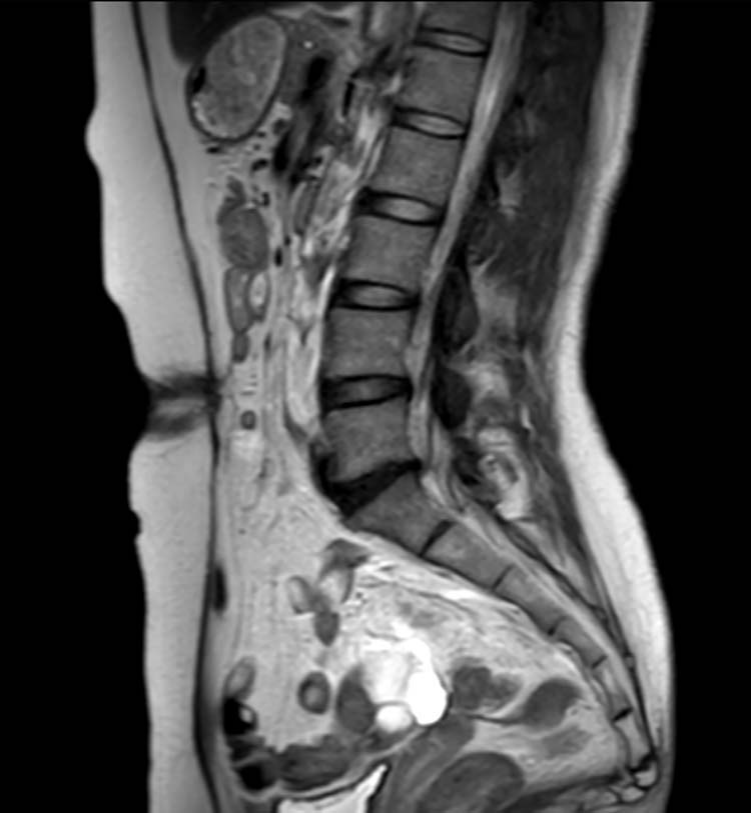
Postoperative MRI. Sagittal view.

**Figure 10:**
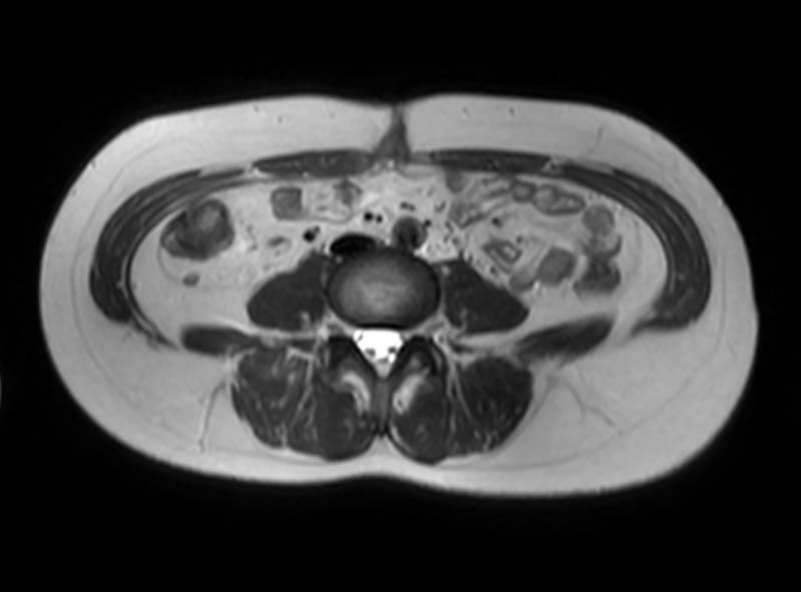
Postoperative MRI. Axial view.

The patient did well after surgery. Follow-up MRI taken 10 days after surgery confirmed the absence of hernia and closure of the fascial defect (Figs 9 and 10).

## DISCUSSION

In recent years, the use of laparoscopic surgery has become widespread. Although PHS is relatively rare, occurring in 0.26–2.5% of patients who undergo laparoscopic surgery [1–4], its incidence is expected to increase with more widespread use of minimally invasive surgical techniques. The causes of PHS include patient factors—such as obesity, older age, wound infection, diabetes and smoking—and surgery-related factors such as operative time, excessive manipulation of the trocar insertion port, port diameter, insertion site and incomplete suturing of the fascia and peritoneum [5–7].

When an abdominal incisional hernia occurs, the options for repair are suturing using native tissue, repair using mesh or a combination. The recurrence rate of simple suture closure in Japan is 9.3–11.4%, higher than the recurrence rate of mesh repair that is 2.4–4.3% [8–10]. However, the use of mesh can create mesh-specific complications. There are reports of mesh infection, extracorporeal mesh exposure, intestinal adhesions and obstruction, and intestinal fistula formation.

Our patient had a 9 × 13-mm fascial defect at a prior port site, which we were able to successfully repair using the VersaOne™ Fascial Closure System. It is indicated for patients with an abdominal wall thickness exceeding 30 mm, in whom reliable suturing of the port site is not easy. With this system, the port site can be reliably closed, including both fascia and peritoneum, while observing the suture from the abdominal cavity with a laparoscope. Because PHS can reportedly develop below the fascia [7], closure of both fascia and peritoneum is important.

With the closure system, the peritoneum at the hernia site is dissected from an intra-abdominal approach, and a skin incision is made at the same site for trocar placement. Since the peritoneum has been dissected in advance, the trocar can be easily placed. The incision is the size of a port: much smaller and less noticeable than the incision required for a normal hernia repair, conferring an aesthetic advantage.

Closure of PHS defects is made more reliable by the use of a Z suture. For example, for a hernia with a diameter of 10 mm, the length inside the hernia is ~3 cm, and the suture length when simply doing knotted suture is ~1.5 cm. The use of two separate knotted sutures or Z sutures may be more reliable for wounds of this length.

To date, this method has been used in ~30 patients without complications or recurrences. In patients with a thick abdominal wall and a PHS about 10 mm in diameter, this system is indicated regardless of the site. It will be necessary to accumulate more cases and follow patients to determine the long-term prognosis after using this surgical technique.

## CONFLICT OF INTEREST STATEMENT

None declared.
